# Microglia in physiological conditions and the importance of understanding their homeostatic functions in the arcuate nucleus

**DOI:** 10.3389/fimmu.2024.1392077

**Published:** 2024-09-04

**Authors:** Mara A. Guzmán-Ruíz, Natalí N. Guerrero Vargas, Ricardo Jair Ramírez-Carreto, Juan Carlos González-Orozco, Bryan Adair Torres-Hernández, Michelle Valle-Rodríguez, Rosalinda Guevara-Guzmán, Anahí Chavarría

**Affiliations:** ^1^ Programa de Becas Post-doctorales, Dirección General de Asuntos del Personal Académico, Universidad Nacional Autónoma de México, Mexico City, Mexico; ^2^ Departamento de Fisiología, Facultad de Medicina, Universidad Nacional Autónoma de México, Mexico City, Mexico; ^3^ Departamento de Anatomía, Facultad de Medicina, Universidad Nacional Autónoma de México, Mexico City, Mexico; ^4^ Unidad de Medicina Experimental “Ruy Pérez Tamayo”, Facultad de Medicina, Universidad Nacional Autónoma de México, Mexico City, Mexico; ^5^ Instituto de Fisiología Celular-Neurociencias, Universidad Nacional Autónoma de México, Mexico City, Mexico

**Keywords:** arcuate nucleus, hypothalamus, microglia, microglial-neuronal interaction, physiological function, obesity

## Abstract

Microglia are highly dynamic cells that have been mainly studied under pathological conditions. The present review discusses the possible implication of microglia as modulators of neuronal electrical responses in physiological conditions and hypothesizes how these cells might modulate hypothalamic circuits in health and during obesity. Microglial cells studied under physiological conditions are highly diverse, depending on the developmental stage and brain region. The evidence also suggests that neuronal electrical activity modulates microglial motility to control neuronal excitability. Additionally, we show that the expression of genes associated with neuron-microglia interaction is down-regulated in obese mice compared to control-fed mice, suggesting an alteration in the contact-dependent mechanisms that sustain hypothalamic arcuate-median eminence neuronal function. We also discuss the possible implication of microglial-derived signals for the excitability of hypothalamic neurons during homeostasis and obesity. This review emphasizes the importance of studying the physiological interplay between microglia and neurons to maintain proper neuronal circuit function. It aims to elucidate how disruptions in the normal activities of microglia can adversely affect neuronal health.

## 1 Introduction

Microglial cells, one of the resident macrophages of the central nervous system (CNS) in mammals, originate from mesodermal yolk sac myeloid progenitors during neurodevelopmental stages. These cells infiltrate the brain during embryogenesis and play a crucial role in neuronal differentiation and maturation (1). Microglia constitute approximately 10% of CNS cells and account for 5 to 20% of glial cells (2, 3).

Although there is no denying that microglia are the first responders against damage and infection, they are also crucial for maintaining brain homeostasis (4), as they can support neurons through several processes such as synaptic pruning, learning, memory, neurogenesis, and neuronal connectivity (2, 5). However, since their role in the brain was first discovered in pathological conditions, studies have paid particular attention to their pathologic role and relegated their physiological function to a dormant sentinel under physiological conditions.

Some studies suggest that microglial activities differ depending on brain region, age, and health status, suggesting that microglia function is heterogeneous, and not restricted to injury-related responses (6, 7). Microglia around various regions of the brain, including the arcuate nucleus (ARC) of the hypothalamus, play a crucial role in maintaining metabolic homeostasis and neuronal communication, so proper development and physiological functioning of microglial cells are essential for preventing metabolic disturbances linked to obesogenic diets. Disruptions in the normal activities of these resident immune cells can contribute significantly to the pathogenesis of obesity and related metabolic disorders (8, 9).

Understanding the mechanisms by which microglia in different brain regions influence metabolic processes is essential for developing targeted therapeutic strategies. This review aims to describe hypothalamic microglial function under physiological conditions, highlighting their critical role in maintaining proper neuronal activity and the physiological responses of ARC. Additionally, it explores the implications of ARC microglia in altered responses to dietary challenges, providing valuable insights into obesity-related neuroinflammatory conditions.

## 2 Brief scope on microglia

Microglia are the primary immunocompetent cells in the brain. As one of the main CNS resident macrophages, microglia play a critical role during physiological conditions. The induction of their immune program has been related to the development of diseases such as Alzheimer’s disease, ischemia, and even obesity (10–13).

Microglial cells present a highly dynamic resting non-immune state to surveil the brain parenchyma constantly; therefore, resting microglia does not mean “inactive” (14). Instead, they maintain baseline motility without inflammation, which consists of their processes’ extension, retraction, and movement. As a result, microglia can survey their environment, clear cellular debris, interact with neurons and other glial components, and remodel the extracellular matrix (15, 16). Furthermore, surveillance motility is highly correlated with morphological modifications such as the number, length, and ramification of their filipodia (17).

As CNS sentinels, quiescent microglia constantly survey the brain parenchyma, searching for damaging signals that may disrupt brain homeostasis (4). Damage-derived stimuli can be detected by microglia throughout four different types of pattern recognition receptors (PRRs): toll-like receptors (TLRs), nod-like receptors (NLR), rig-like receptors (RLR), and c-type lectin receptors (CLR) (11, 12, 16, 17). Activation of these receptors can initiate a multifaceted response in microglia, including phagocytosis, production of cytotoxic molecules, and promotion of signals that repair and restore brain tissue (17).

The microglial cytotoxic response is triggered by exposure to pathogen‐derived antigens like lipopolysaccharide (LPS), dying cells, or the accumulation of misfolded proteins in the extracellular matrix (4, 18). This function implies the production of pro-inflammatory cytokines such as tumor necrosis factor-α (TNFα), interleukin-1β (IL-1β), IL-6, IL-12, IL-23, nitric oxide (NO), reactive oxygen species (ROS), matrix metalloproteinases (MMPs), chemokines and redox molecules (e.g., NADPH oxidase or iNOS), among others. They also express other molecules like scavenger receptors (e.g., macrophage receptors with collagen structure), co-stimulatory proteins like the cluster of differentiation 40 (CD40), and the major histocompatibility complex II (MHC-II) (12, 18, 19). A similar pro-inflammatory response has been observed in the absence of infection, for example, during brain trauma, cell degeneration, or chemical exposure (19).

Microglia may also have a neuroprotective capacity characterized by the production of anti-inflammatory cytokines such as IL-4, IL-13, IL-10, transforming growth factor beta (TGF-β), and neurotrophic factors like vascular endothelial growth factor (VEGF), and epidermal growth factor (EGF) (12). After the initial pro-inflammatory immune activation, microglia gradually acquire a neuroprotective function to promote tissue repair, neuronal survival, and the reconstruction of the extracellular matrix (12, 20, 21).

Different stimuli, such as physical trauma, infection, systemic inflammation, tumor, ischemia, and neurodegeneration, may activate microglial immune functions. In the present review, we will not employ the terms M1 and M2 activated microglia since these profiles were coined based on studies exposing *in vitro* microglia to immune challenges such as LPS or combinations of pro-inflammatory cues, which are not replicated *in vivo* (21, 22).

Besides these widely studied immune functions, microglial cells might perform other tasks without any immune challenge. These tasks might be determined by different factors such as developmental stage, brain region, sexual dimorphism, and even animal species (22–27). The following sections will discuss microglia’s less-described physiological functions.

## 3 Microglia diversity in physiological conditions

Microglial cells are far more complex under physiological conditions. Despite the countless publications assessing the role of microglia under many pathological conditions, we still do not know the exact implications of microglial function under physiological conditions beyond the embryonic stages, such as synapse pruning, axon myelination, and trophic factor secretion for neurogenesis (28, 29).

In the yolk sac, early progenitor c-kit^+^ lineage cells give rise to microglia Cx3CR1^+^ colonizing brain tissue and accompanying neural precursors during neurodevelopmental stages (30). Afterward, during embryogenesis and early postnatal life, microglia respond to brain microenvironment changes (31). They can engulf presynaptic inputs and phagocyte apoptotic cells, pruning synapses, guiding neurogenesis, and refining synaptogenesis and myelin formation (32, 33). This microglial developmental role is thought to be associated with their immune function.

Microglia can trigger neuronal apoptosis by secreting TNFα, reactive oxygen species, and glutamate, among other factors (34), to initiate cell death programs in stressed or damaged cells to eventually phagocyte them through signaling pathways that include triggering the receptor expressed on myeloid cells 2 (TREM2), MER proto-oncogene, tyrosine kinase (MERTK), and milk fat globule EGF and factor V/VIII (MFG-E8) (35).

In addition, microglia maintain CNS homeostasis, and alterations in their function caused by deletions or mutations in TREM2 or the colony-stimulating factor 1 receptor (CSF1R) cause neurodegeneration or leukodystrophies, respectively (36–38).

During development, microglial cells contact synapses through CX3CR1 and the P2Y12 receptor (P2Y12R) by sensing and responding to neuronal activity. After the induction of long-term potentiation (LTP) in the hippocampus, ramified CX3CR1^+^ cells increase, thus establishing more contact with dendritic spines (39). These effects in LTP-induced microglial dynamic were absent after the administration of an NMDA antagonist. Importantly, microglia-spine contacts are rare and brief during basal synaptic hippocampal activity, suggesting that microglia sense high-frequency neuronal activity as indicated by the associations observed between dendrites, somas, and axons in the healthy brain (40, 41). Notwithstanding, it is unknown which signals may induce microglia-neuron associations, although glutamate has been a feasible candidate.

Embryonal and neonatal microglia express a highly characteristic transcriptomic profile, which differs from those encountered in adult microglia (6, 26), which might reflect their accelerated activity during these developmental stages. Hammond et al. identified transcriptionally distinct microglial subpopulations along distinct developmental ages, embryonic day 14.5 (E14.5), early postnatal day 4 or 5 (P4/5), late juvenile stage at postnatal day 30 (P30), adulthood at postnatal day 100 (P100), and old age at postnatal day 540 (P540), where the greatest microglial diversity was found at E14.5 and P4/5. Interestingly, the transcriptomic profile in these early stages completely differed from the microglia in old animals P540 and injured brains (6).

As previously mentioned, early postnatal brain microglia cells are involved in regulating axonal growth and fasciculation and in the refinement of synaptic circuits. These early-life microglial cells express high levels of genes like the insulin-like growth factor 1 (*Igf1*), which is an essential embryonic growth factor for myelinogenesis (42), the glycoprotein non-metastatic melanoma protein B (*GpnmB*), that is thought to provide neuroprotection (43), galectin-1 (*Lgals1*) and galectin-3 (*Lgals3*), well know immuno-modulators known to deactivate cytotoxic microglia (44) and the lysosomal markers; lysosomal-associated membrane protein 1 (*Lamp1*) (45) and (*Cd68*) (6, 46).

In the same study, eight microglial subpopulations were defined during early developmental stages by analyzing their specific transcriptional programs defined by the expression level of the following genes: arginase 1 (*Arg1*) (47), ribonucleotide reductase M2 (*Rrm2*) (48), ubiquitin-conjugating enzyme E2C (*Ube2c*) (49), centromere protein A (*Cenpa*) (50), fatty acid binding protein 5 (*Fabp5*) (51), osteopontin (*Spp1*) (52), heme oxygenase 1 (*Hmox1*) (53), and membrane-spanning 4-domains, subfamily A, member 7 (*Ms4a7*) (54), suggesting that each microglial subpopulation may perform specific functions (see **Table 1**).

**Table 1:** Genes defining microglial subclasses along developmental stages.

Developmental stage	Up-regulated genes	Function	References
**Embryonal and early postnatal**	Insulin-like growth factor 1 (*Igf1*)	Pleiotropic molecule with neurotrophic and immunomodulatory functions	Wlodarczyk et l. (42)
	Glycoprotein non-metastatic melanoma protein B (*GpnmB*)	Neuro-protection	Satoh et al. (43)
	Galectin-1 (*Lgals1*) and galectin-3 (*Lgals3*)	Immuno-modulators that deactivate cytotoxic microglia	Starossom et al. (44)
	Lysosomal-associated membrane protein 1 (*Lamp1*)	Glycoprotein expressed in lysosomal membranes	Barrachina et al. (45)
	Cluster of differentiation 68 (*Cd68*)	Receptor expressed in lysosomes	Hammond et al. (6), Kettenmann et al. (46)
	Subpopulation defining genes	Function	References
	Arginase 1 (*Arg1*)	Metalloenzyme that inhibits the production of nitric oxide (NO) usually expressed in anti-inflammatory microglia	Cherry et al. (47)
	Ribonucleotide reductase M2 (*Rrm2*)	Small subunit in ribonucleotide reductases, that participates in nucleotide metabolism and catalyzes the conversion of nucleotides to deoxynucleotides	Zuo et al. (48)
	Ubiquitin-conjugating enzyme E2C (*Ube2c*)	Enzyme that is part of an intrinsic inhibitory mechanism, required for the disintegration of mitotic cyclins and securins after spindle assembly during mitosis	Kumar et al. (49)
	Centromere protein A (*Cenpa*)	Part of the centromere proteins involved in epigenetic regulation of centromeres	de Rop et al. (50)
	Fatty acid binding protein 5, epidermal (*Fabp5*)	Member of the FABP family with a high affinity for docosahexaenoic acid (DHA),a molecule that is able to reduce the release of pro-inflammatory molecules from primary murine microglia	Low et al. (51)
	Osteopontin (*Spp1*)	Matricellular protein secreted by every CNS cell that signals to CD44 triggering pro-inflammatory responses in macrophages. Associated to Tract-Associated Microglia (ATM) of the early PN brain	Rosmus et al. (52)
	Heme oxygenase 1 (*Hmox1*)	Catalyzes the oxidation of heme to biliverdin and carbon monoxide	Deininger et al. (53)
	Membrane-spanning 4-domains, subfamily A, member 7 (*Ms4a7*)	Membrane protein expressed in anti-inflammatory microglia with a pro-oncogenic role in glioblastoma	Ni et al. (54)
**Juvenile and adult**	Transmembrane protein 119 (*Tmem119*)	Membrane type-I protein with amyloid precursor protein-like structure.	Ruan et al. (55)
	Selectin P ligand (*Selplg*)	Adhesion molecule critical for cell migration and chemotaxis.	Rossi et al. (56)
	Purinergic G Protein-coupled receptor Y13 (*P2ry13*)	Purinergic receptor involved in motility of microglial processes to focal damage sites.	Kyrargyri et al. (57)
	Colony-stimulating factor 1 receptor (*Csf1r*)	Tyrosine-kinase transmembrane receptor that regulates microglial homeostasis.	Hu et al. (38)
	C-X3-C motif chemokine receptor 1 (*Cx3cr1*)	Chemokine receptor that binds to fractalkine ligand which is associated with crosstalk between neurons and microglia.	Ho et al. (58)
	Maf family protein B (*MafB*)	bZIP transcription factor involved in negative regulation of GM-CSF signaling and promotes an anti-inflammatory phenotype.	Koshida et al. (59)
	Myocyte enhancer factor 2A (*Mef2a*)	Protein involved in inflammatory gene expression and its modulation.	Cilenti et al. (60)
	Activator protein-1 family transcription factors Jun (*Jun*) and Fos (*Fos*)	Transcription factors which maintain microglia in surveilling phenotype.	Holtman et al. (61)
	Complement C1q fraction a (*C1qa*)	Polypetide A from C1q protein involved in the complement enzymatic cascade reactions.	Fonseca al. (62)
	Early growth response-1 (*Egr1*)	Oxidative stress-sensitive transcriptional factor involved in proinflammatory responses and neuronal plasticity.	Yu et al. (33)
	Prostate transmembrane protein androgen induced 1 (*Pmepa1*) and cluster of differentiation 14 (*Cd14*)	Protein core that modulates immune reactions.	Javanmehr et al. (63)
**Aged**	Aged Subtype OA2		
	Galectin-3 (*Lgals3*)	Immuno-modulators that deactivate cytotoxic microglia	Starossom et al. (44)
	Cystatin F (*Cst7*)	Is amongst the most robustly upregulated genes in diseased associated microglia	Daniels et al. (64)
	Chemokine *Ccl4 or* macrophage inflammatory protein-1b (Mip-1b)	Chemokines regulate the recruitment and activation of circulating and resident immune cells in all tissues,	Kremlev et al. (65)
	Chemokine Ccl3		
	Interleukin 1 beta (Il1b)	Pro-inflammatory cytokine	Liu et al. (66)
	Transcriptional regulator DNA binding protein inhibitor 2 (Id2)	ID2 represses basic helix-loop-helix transcription factors and is involved in the differentiation of immune cells	Holtmann et al. (61)
	Activating transcription factor 3 (ATF3)	Negative regulator of Il6 and Il12b transcription	Holtmann et al. (61)
	Aged Subtype OA3		
	Interferon induced transmembrane protein 3 (Ifitm3)	Member of the interferon-inducible transmembrane family, that serves as a molecular mediator between amyloid pathology and neuroinflammation.	Harmon et al. (67)
	Receptor transporter protein 4 (*Rtp4*)	Member of the RTP family known to negatively regulate of IFN-I responses	He et al. (68)
	2 -5 oligoadenylate synthetase-like 2 (*Oasl2*)	Involved in the innate antiviral response. OASL enhances DNA virus replication by binding to the DNA sensors, inhibiting IFN induction.	Ghosh et al. (69)

Meanwhile, there is varying information about transcriptional profiles regarding juvenile and adult-derived microglial states. Some authors claim these are less diverse, identifying only three or two distinct subpopulations. These groups were sorted by their differential gene expression rather than specific genes (6, 70). In contrast, other studies using genome-wide chromatin and expression profiling, combined with single-cell transcriptomic analysis in the cortex, hippocampus, and spinal cord, describe a global gene expression pattern for adult microglia. It highlights Selectin P ligand (*Selplg*) (56), Prostate transmembrane protein androgen-induced 1 (*Pmepa1*) (63), cluster of differentiation 14 (*Cd14*) (63), Activator protein-1 family transcription factors Jun (*Jun*) and Fos (*Fos*) (61), Myocyte enhancer factor 2A (*Mef2a*) (60), and Maf family protein B (*MafB*) (59) as genes strongly correlated with adult microglia in physiological conditions (**Table 1**) (61, 71). Further studies have determined that markers like TMEM119, P2ry12, and P2ry13 are up-regulated in the mature brain (55, 57, 72).

Furthermore, the microglia derived from aged mice present transcriptional profiles with the up-regulation of genes involved in immune activation and the development of neurodegenerative diseases (64–69, 73, 74) (**Table 1**). All these data suggest that microglial function under physiological conditions varies according to the developmental stage.

Healthy microglia also present brain-region-associated transcriptional profiles. Masuda et al. also performed single-cell RNA sequencing (scRNA-seq) of microglia in homeostatic conditions of multiple anatomical regions of the CNS of embryonic, juvenile, and adult mice. Authors found two main clouds differentiating embryonic from juvenile and adult from postnatal microglia. Within the cloud derived from postnatal mice, t-SNE analysis showed six sub-clusters in postnatal microglia, while juvenile and adult microglia only presented four. Each sub-cluster had specific transcriptional patterns suggesting the existence of different microglial subclasses during both embryonic and juvenile/adult stages (27).

Similarly, the analysis of different regions within either embryonic, postnatal, and juvenile/adult mice revealed that each anatomical area of the CNS presented a regional distribution of transcript expression, denoted as microglial molecular signature, evidencing the existence of spatiotemporal microglial subclasses (27).

Later, Zheng et al. reported differences in the molecular signature between cortical and spinal microglia. This study characterized three distinct microglial clusters in the cortex and two in the spinal cord (7). Within cortical microglia, two sub-clusters exhibiting different expression levels of homeostatic genes were defined as homeostatic microglia 1 and 2 (HOM-M1 and HOM-M2), both differing in the expression of genes coding ribosomal proteins, molecular pathways involved in the establishment of homeostatic functions, among others (7). Furthermore, another subtype that expressed immune genes was identified as inflammatory microglia (IFLAM-M). This IFLAM-M was less represented in the cortex, while it constituted 45% of the spinal microglia in two-month-old mice; this percentage varied along the lifespan, suggesting that the immune function of these cells is age-related.

Interestingly, cortical microglia maintained a relatively low proportion of IFLAM-M, suggesting that the expression of inflammatory genes is more restrained in the cortex than in the spinal cord (7). Hammond et al. reported that the microglia of young mice are more heterogeneous and that the inflammatory pathways were mainly enriched in aged individuals (6). All these studies suggest that microglial molecular signatures differ according to age, brain region, and health status, indicating that these cells’ function is not homogeneous.

As dynamic cells, besides constantly surveilling the milieu, microglia are thought to detect neural electrical activity (40, 41, 75). Nimmerjahn et al., using *in vivo* two-photon microscopy, determined that microglial processes were significantly motile, where their filopodia experienced several extend-withdraw cycles under physiological conditions (14, 75). This motility process directs microglial podocytes to establish transitory contacts with dendritic spines in healthy mice’s somatosensory and visual cortexes in response to neuronal sensory stimulation (40). Conversely, sensory deprivation results in filopodial retraction, thus reducing the number of neuronal contacts in the visual cortex *in vivo* (40).

The exact signal microglia detect that redirects their processes toward activated neurons is unclear. Neuronal mitochondrial activity, induced by neuronal electrical activation, rapidly triggers the establishment of the microglia-neuron junction and is blocked by inhibition of P2Y12 receptors for adenosine 5’diphosphate (ADP), suggesting that the communication established through this receptor might allow the transitory junction observed between neurons and microglia (76).

Stimulation of hippocampal CA3 neurons that project to CA1 pyramidal cells through the Schaffer collateral pathway in mice results in an increase in microglial Ca^2+^ in early postnatal hippocampal CA1; this effect depends on neuronal action potentials since tetrodotoxin (TTX) administration significantly reduce microglial Ca^2+^ influx (77).

Microglial cells’ ability to detect and redirect their filopodia in response to neuronal electrical activity might be crucial to preserving a homeostatic neuronal firing rate (78–80). Blocking microglial capacity to redirect themselves toward firing neurons leads to the hyper-synchronicity of cortical circuits in response to sensory stimulation (79).

Although microglial transcriptional profiles vary depending on brain region, only a handful of studies approach region-specific microglial function in homeostatic conditions. Hypothalamic microglia have been approached to understand their implication in neuroinflammation resulting from consuming a high-fat diet (HFD), considering microglial cells as mere sentinels instead of active participants of the hypothalamic circuits’ physiology. In the following sections, we will explore the possible implications of hypothalamic microglia for its daily homeostatic function.

## 4 Brief scope of the medio-basal hypothalamic arcuate nucleus

Two primary problems in studying ARC microglial cells are that many of the models employed are not ARC-specific and that the studies do not focus on what this cell type does under physiological conditions.

**Figure 1:**
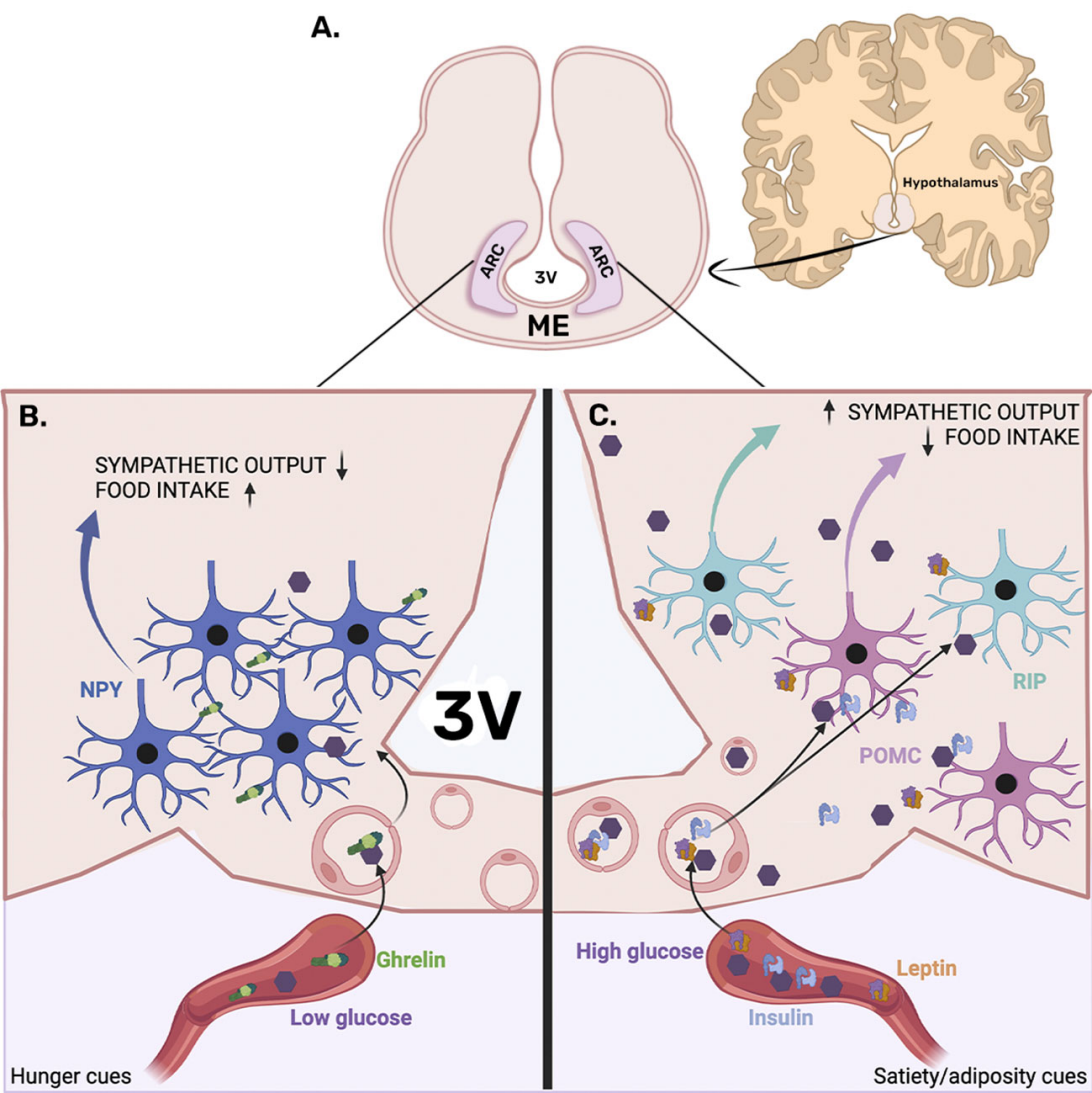
Arcuate nucleus location and neuronal organization. **(A)** The arcuate nucleus (ARC) is located in the medio-basal hypothalamus, adjacent to the third ventricle (3V), attached to the median eminence (ME). **(B)** The left panel presents ARC under fasting conditions, where hunger cues like low glucose levels and ghrelin secreted by the stomach can access the ARC parenchyma through the fenestrated vascularization of the median eminence, which eventually reaches the orexigenic neuropeptide Y (NPY) expressing neurons. NPY activation reduces sympathetic output, which consequently decreases energy expenditure. In addition, NPY neurons promote food intake. **(C)** The right panel presents the entrance of satiety/adiposity signals like insulin and leptin, which are known to activate pro-opiomelanocortin (POMC), promoting energy expenditure and inhibiting food intake. Furthermore, leptin is known to activate RIP neurons known to increase energy expenditure by promoting brown adipose tissue-induced thermogenesis.

Comparative studies in mammals suggest that species-specific developmental programs link anatomy, cellular differentiation, and gene expression to create hypothalamic ‘modules’ that can be gained or lost through evolution (81). Similar hypothalamic nuclei found in rodents and humans indicate possible homology at both anatomical and functional levels (82). In humans, the arcuate nucleus (ARC) is located in the medio-basal hypothalamus, adjacent to the third ventricle and attached to the median eminence (ME) (**Figure 1**). It is considered a circumventricular organ (83). This location allows cerebrospinal fluid (CSF) and blood-borne cues to enter the ARC parenchyma since the ME is highly vascularized with fenestrated capillaries originating from the hypophyseal portal system (84). Therefore, metabolic signals, like plasmatic glucose, triglycerides, leptin, insulin, and ghrelin, can freely reach and modulate the neuronal activity of the ARC (85–90).

In response to metabolic status, ARC neurons modulate both aspects of energy balance, food intake, and energy expenditure. In fasting conditions, plasmatic glucose levels decrease, and the hunger hormone ghrelin is secreted in response to gastric emptying. Both low glucose and ghrelin are known to activate ARC neuropeptide Y (NPY) and agouti-related peptide (AgRP) neurons to promote food intake and diminish energy expenditure (85). NPY and AgRP are also glucose-inhibited neurons since food intake-induced blood glucose elevations inhibit them from ceasing their orexigenic function and preventing overeating (91–94).

ARC-NPY afferences to secondary hypothalamic nuclei reduce the sympathetic output to the brown adipose tissue (BAT), a region that increases energy expenditure for heat production (95). In fasting conditions, NPY-mediated BAT thermogenic inhibition is observed (96). In addition, activation of the ARC-NPY neurons suppresses the sympathetic output of pre-autonomic pathways, thus decreasing blood pressure (97). Similarly, activation of the ARC-AgRP neurons promotes insulin resistance by inhibiting the sympathetic output that activates BAT glucose uptake (98). Furthermore, NPY projections to the paraventricular nucleus (PVN) are known to induce food intake (99, 100) (**Figure 1**).

Conversely, after food ingestion, satiety cues such as elevated glucose and insulin levels increase, and adipose-derived signals like leptin inhibit the NPY/AgRP neuronal activity and increase the firing rate of the pro-opiomelanocortin (POMC) and cocaine amphetamine-related transcript (CART) neurons, known to inhibit food intake and to increase energy expenditure (85, 101, 102, 103) (**Figure 1**). Additionally, ARC-GABAergic-RIP neurons modulate the sympathetic outflow, promoting energy expenditure by the noradrenergic stimulation of BAT-mediated thermogenesis (104) (**Figure 1**).

Although the neurons in the ARC are critical players in modulating the outputs that regulate several bodily functions involved in controlling metabolism, recent studies have demonstrated that ARC glial populations are also crucial for maintaining their function (105–107). Specifically, hypothalamic microglia maintain ARC neuronal function and are essential for developing metabolic diseases such as obesity (108, 109).

As previously mentioned, microglia are highly active cells under both physiological and pathological conditions; however, the ARC microglia have mainly been implicated in the hypothalamic inflammation resulting from obesity, although a few studies suggest that ARC microglia are constantly surveilling ARC neuronal function.

## 5 Microglia physiological functions in the ARC

The traditional view of microglia as mere phagocytic cells responsible for eliminating synapses, dead or apoptotic cells, and cellular debris is overly simplistic. Microglia play a crucial role in synaptic formation, reorganization, maturation, and neurogenesis. They achieve this through direct contact, the release of soluble factors, the engulfment of synaptic structures, and various microglia-neuronal signaling pathways during the remodeling of brain circuits. This dynamic process continues throughout life, allowing the brain to adapt to its ever-changing microenvironment. In addition, proper development and maintenance of hippocampal and hypothalamic neuronal circuits rely heavily on functional microglia (110). It has been suggested that the physiological implication of ARC microglia modulates feeding behavior and energy balance.

One piece of evidence is that the functional microglial marker, such as the cluster of differentiation 68 (CD68), a vesicle marker, changes according to the time of the day without immune stimulation (111). CD68, a member of the lysosome-associated membrane protein (LAMP) family, participates in vesicle mobilization, a process found in macrophages during phagocytosis, lysosome digestion, and solute secretion. The daily non-immune associated changes in CD68 expression in the ARC could be involved in any vesicle-forming process that repeats itself every 24 h (69).

**Figure 2:**
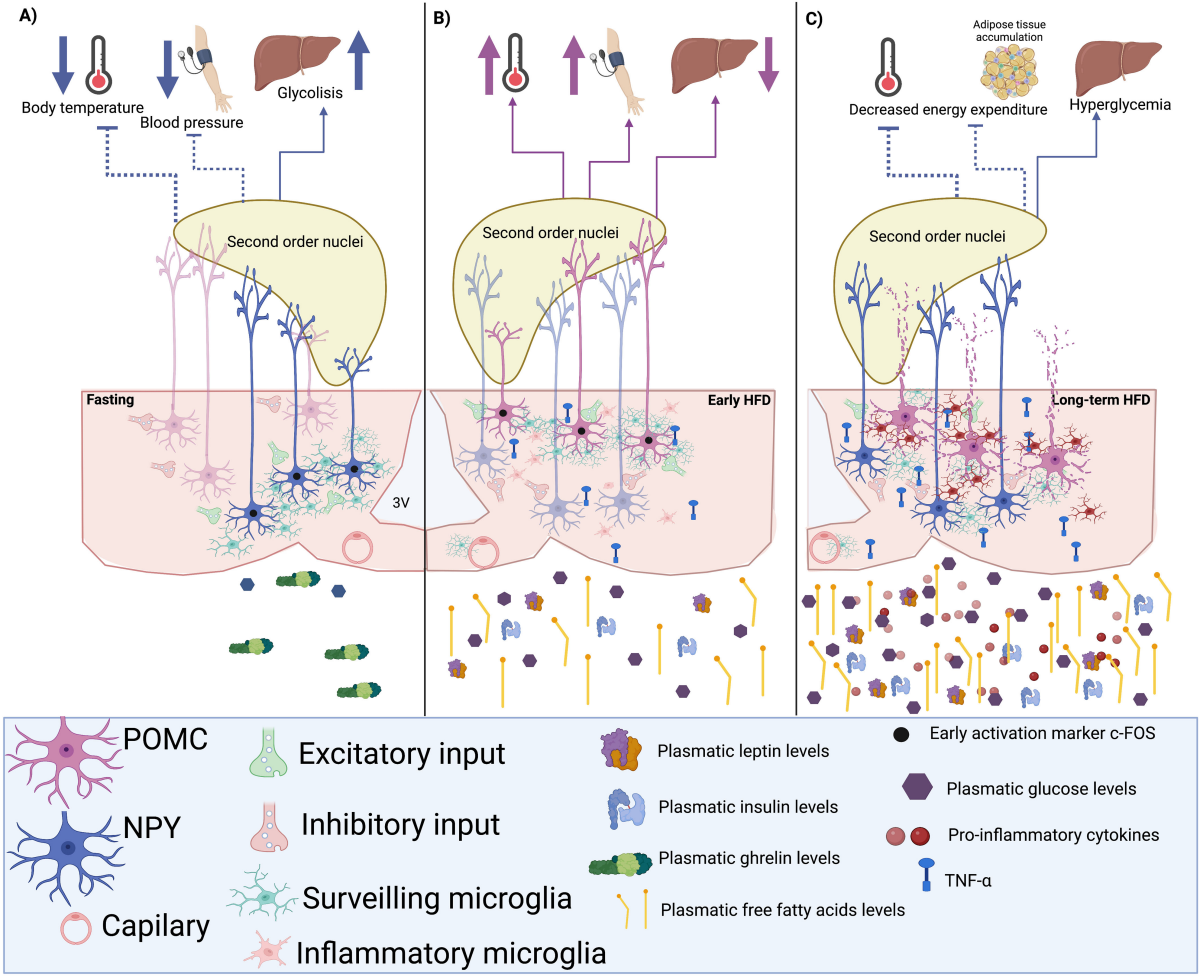
Hypothetic microglia-neuron circuit in the arcuate nucleus (ARC). **(A)** A hypoglycemic state induces the activation of neuropeptide Y (NPY) neurons and the rearrangement of microglial cells during fasting, increasing excitatory inputs to NPY neurons and their firing rate. Consequently, NPY output would inhibit the neuronal activity of second-order nuclei like the paraventricular nucleus (PVN), decreasing the sympathetic tone and reducing energy expenditure by inhibiting heat production and decreasing blood pressure. **(B)** The short-term consumption of a high-fat diet (HFD) could induce the rearrangement of microglia towards POMC neurons, raising their firing rate in response to glucose, insulin, and leptin to promote the activation of sympathetic outputs that improve energy expenditure and decrease hepatic glucose production. **(C)** When the consumption of an HFD is chronic, the inflammation of the adipose tissue induces the presence of pro-inflammatory circulating signals that reach and activate ARC microglia. The inflamed hypothalamus would cause neuronal damage, especially in POMC neurons and not in NPY cells, which may contribute to low metabolic rates and probably hyperglycemia.

Another ARC microglial action observed by Winkler et al. is the rearrangement of these cells in juxtaposition to NPY-activated neurons in response to a drop in plasmatic glucose levels elicited either by fasting or an insulin i.v. administration (112). This effect was inhibited by an intracerebroventricular (i.c.v.) minocycline microinjection, a microglial inhibitor (112). Furthermore, minocycline i.c.v. administration increased the counterregulatory glucose production in response to a hypoglycemic stimulus, indicating that ARC-NPY (glucose-inhibited) neuronal activity is sensed by microglial cells, thus modulating these cells’ response to hypoglycemia (**Figure 2A**).

In another study, Jin and collaborators demonstrated that stimulating the microglial TLR2 through an i.c.v. Pam3CSK4 administration rapidly triggers the rearrangement of these macrophages toward ARC-POMC neurons. This effect was associated with changes in the percentage of synaptic inputs contacting POMC neurons, increasing their excitatory inputs and raising their excitatory activity, ultimately resulting in anorexia and increased body temperature (113). In the same study, the minocycline-mediated microglial inhibition successfully prevented the observed anorexia and thermogenesis (113). Also, stimulation of microglial TLR4 promotes an excitatory response in POMC neurons, whereas inhibiting AgRP/NPY neurons (114). These data demonstrate that ARC microglia not only sense ARC neuronal activity but may also regulate neuronal excitability and their output, consequently modulating the biological effect of ARC neuronal populations.

In anorexic humans and rodent models, Iba1 brain expression and enrichment of microglia genes are increased (115, 116). Also, the administration of deoxynivalenol (DON), a compound known to induce microglial inflammatory function in circumventricular organs such as the ME, causes anorexia. Interestingly, PLX3397 microglial depletion enhanced DON sensitivity, causing food intake inhibition in response to non-anorectic DON doses and increased neuronal activation in the ARC and the PVN (117).

Furthermore, ARC microgliosis has been described in the early phases of pancreatic ductal adenocarcinoma, which has been associated with cachexia by altering the communication between POMC ARC neurons and the PVN (118). CSF1-R-mediated microglia depletion accelerates the cachexia onset and increases anorexia (118). These data suggest that microglia is a crucial modulator of ARC neuronal excitability, and its respective outputs control feeding behavior. Furthermore, microglial response to metabolic and immune challenges might contribute to preventing energy imbalance.

Other studies have also inhibited or depleted microglia and observed critical metabolic effects. Eight-week-old C57BL/6 mice subjected to whole-body irradiation received bone marrow transplants from green fluorescent protein (GFP)-transgenic C57BL/6 mice with a deletion of the BDNF gene, resulting in higher body weights. However, the establishing site of these cells is preferentially the PVN instead of the ARC (119). Likewise, conditional ablation of microglia in *Cx3cr1*-Dtr rats reduced food intake and energy expenditure (120). Interestingly, increasing brain CX3CL1 levels prevented diet-induced obesity in male mice (121), suggesting that the contact-dependent relationship established between microglia and neurons is crucial for maintaining energy homeostasis.

Campbell et al. performed a single-cell analysis of the ARC-ME of mice fed a normocaloric diet or an HFD, thus presenting a transcriptional census of these areas. They identified 50 distinct ARC-ME cell populations, such as tanycytes, leptin-sensing neurons, AgRP, and POMC subtypes, among others (122). Since ARC microglial cells respond to NPY and POMC neuronal activity, the mechanisms that connect these cellular populations might be deeply affected by an HFD.

The CD200-CD200R1 system is an *in vivo* “Off” signal that comprises the transmembrane glycoprotein ligand CD200, expressed by neurons and endothelial cells, and its receptor CD200R1, which is expressed in myeloid cells like microglia (123). Studies have demonstrated an up-regulation in CD200 in the neocortex, hippocampus, and striatum of the R6/1 transgenic mouse model of Huntington’s disease (HD), with unaltered expression in CD200R1 (124). This data indicates a counter-regulatory neuronal mechanism to maintain the neuronal–microglial communication to sustain neuronal function under a pro-inflammatory condition like HD or an HFD. The relationship established between microglia and the ARC-neuronal circuits has been widely studied during obesity; the following section will discuss how microglial non-physiological function in response to high-fat, high-carbohydrate diet consumption and the resulting low-grade inflammation during obesity may impair the ARC microglial-neuronal relationship therefore, de-regulating metabolic homeostasis.

## 6 Microglial response to obesity and its possible implications for ARC neuronal activity

Obesity is a pro-inflammatory state characterized by the hypertrophy and hyperplasia of the white adipose tissue (WAT), in which adipocytes secrete pro-inflammatory cytokines and chemokines, thus maintaining a mild inflammatory tone in the body for prolonged periods (125). Not only do cytokines and chemokines have inflammatory roles in obesity, but WAT can also react by producing and secreting biologically active substances as hormones or peptides, which are termed “adipokines” (126) that contribute to the obesity-derived chronic low-grade systemic pro-inflammatory condition, also known as “metainflammation” (127). The hypothalamus responds to the low-grade inflammation observed during obesity by further expressing pro-inflammatory cytokines (108, 128–131) that eventually impair hypothalamic insulin and leptin sensitivity (132, 133).

As previously mentioned, the ARC is a sensory region. Thus, its distinct cell populations can detect and respond to blood-borne circulating metabolic and inflammatory signals. The consumption of high-fat and high-carbohydrate diets increases plasmatic free fatty acids (134, 135), which, as they tend to accumulate in the white adipose depots, can cause inflammation and, eventually, neuroinflammation (136, 137), by initiating an innate immune response elicited in glial cells (138, 139).

Specifically, microglia are the first to respond to dietary saturated fatty acids, promoting lipid-induced neuronal stress, hypothalamic inflammation, leptin and insulin resistance, and hyperphagia in mice (140, 141). Furthermore, i.c.v. infusions of saturated and polyunsaturated fatty acids induce the expression of neuroinflammatory markers (131, 142–144), alter autophagic protection from cellular stress (145), and increase endoplasmic reticulum stress responses to unfolded proteins (130, 146).

In diet-resistant mice fed an HFD for only 1-2 weeks, which is not enough time to develop increased adiposity nor metabolic impairments, the number of inhibitory synapses directed towards the ARC-POMC neurons was elevated in the non-diet-resistant mice (control) that eventually became obese. Later, when HFD non-obesity resistant mice became obese, the number of inhibitory synapses associated with ARC-POMC neurons was significantly increased (147). As mentioned, POMC neurons inhibit food intake and promote energy expenditure by regulating the pathways controlling autonomic outputs. This suggests that microglial response during the first stages of HFD consumption highly regulates the synaptic inputs that modulate neuronal excitability.

Paradoxically, Douglass et al. recently showed that microglial inflammatory function during an HFD consumption enhances glucose physiological responses regardless of inducing adiposity (148) and preventing microglial IKKb signaling pathway in response to an HFD prevents obesity but impairs glucose tolerance (148). Furthermore, hypercaloric diets stimulate microglial TLR4, which responds to lipids (149, 131), thus inducing TNFα secretion, inhibiting NPY/AgRP neuronal activity (150) and increasing POMC neuronal excitability (114, 151).

Thaler et al.’s observations could explain this paradoxical effect of microglial pro-inflammatory response in glucose tolerance since a significant rise in hypothalamic pro-inflammatory gene expression was detected after only three days of consuming a hypercaloric diet. This gene profile was associated with increased ARC microglial markers, suggesting that the Iba1 increase within the first days of an obesogenic diet might reflect an increase in microglial function to counteract the excess in energy intake (108). This hypothesis is supported by the Douglass et al. report, where microglial activation promoted parasympathetic insulin secretion (148).

Furthermore, high-fat intake increases palmitate levels in cerebrospinal fluid and triggers a wave of microglial metabolic activation characterized by mitochondrial membrane activation, fission, and metabolic skewing towards aerobic glycolysis (152). Also, a hypercaloric diet increases microglial lipoprotein lipase (LPL) expression, an enzyme relevant for microglial lipid uptake. Mice lacking microglial-LPL are prone to become obese when fed both a control and an HFD (153), implying that ARC microglial immune activity might be part of the normal responses evoked by hypercaloric diets to prevent the metabolic impairments caused by the increased glucose disponibility before the development of obesity (**Figures 2B, C**). Further studies should assess if microglia can adapt or change their morphology, biomarkers, and cytokine secretion profile to the neuronal activity elicited by the consumption of HFD before developing a pro-inflammatory state.

Previous studies have suggested that defective regulation of POMC neurons precedes HFD inflammation and obesity development (154). RNA-seq studies of the POMC neurons of obese mice unveil an enrichment in apoptosis, chemokine signaling, and sphingolipid metabolism pathways, suggesting that an obesogenic diet causes POMC apoptotic neuronal loss (155). As previously mentioned, TNFα increases POMC neuronal excitability (151) and induces elevated blood pressure via a central mechanism involving sympathetic activation (156). This hypothesis is supported by the observation that during obesity, POMC neurons present a higher percentage of microglial contacts (151), suggesting that microglial TNFα constant release during obesity might affect POMC activity and even induce neurotoxicity since there is a significant decrease in the number of POMC neurons after chronic feeding with a high fat and carbohydrate diet (151). These observations indicate that TNFα secreting microglia may increase ARC-POMC neuronal activity, altering their autonomic output.

In contrast, postmortem studies in type II diabetic patients have shown an increase of NPY neurons in the ARC (157), implying that inflammatory signals’ effect on NPY neurons does not promote excitatory inputs, hence not hindering their survival. In fact, studies have demonstrated that long-term palmitate and TNFα exposure promotes NPY mRNA transcription (158); however, King et al. reported NPY neuronal inhibition after IL-1ß, IL-6, and TNFα administrations (159) (**Figure 2C**). Future studies of the exact effect of cytokines in NPY cell cycle programs or survival markers should be performed to understand how they survive an HFD while POMC neurons are significantly reduced.

Moreover, microglia trigger a complex hypothalamic immune response to dietary excess. After one week of a hypercaloric diet, mice presented a monocytic infiltration in the ARC; this infiltration was absent in control-fed mice (160). However, circulating monocyte recruitment is not the primary mechanism for microgliosis and its pro-inflammatory response during the development of obesity (161). Valdearcos et al. also defined two ARC microglial subpopulations: CX3CR1+/P2Y12+ and CX3CR1+/TMEM119+ microglial cells. After the HFD, GFP+CD68+ bone-marrow-derived cells were detected in the ARC; these cells were neither TMEM119+ nor P2Y12+, indicating their myeloid origin. These infiltrating cells arrive after the inflammatory response elicited by the hypothalamic parenchyma (162, 163), consequently recruiting further immune cells from the periphery, such as neutrophils, lymphocytes, and natural killer T cells, into the hypothalamus. Also, dendritic cell migration could be associated with the obesity-induced myeloid cell hypothalamic monocytic invasion contributing to hypothalamic inflammation (160).

Lee et al. demonstrated that perivascular macrophages secrete inducible nitric oxide synthase (iNOS) in mice fed an HFD, contributing to BBB leakage and increased vascular permeability in the hypothalamic parenchyma (164), probably facilitating peripheral immune cell infiltration. Likewise, hypothalamic infiltrated myeloid cells and perivascular macrophages secrete the VEGF (165), contributing to blood-borne metabolic signals’ increased permeability in the ARC (166). These data indicate that non-microglial macrophages are crucial for maintaining hypothalamic circuit homeostasis.

Since metainflammation has been correlated with a “low-grade” chronic microglial activation state, hypercaloric diets have been associated with hypothalamic dysfunction, including loss of synapses, lack of response to metabolic hormones, disturbed organelles function, and cell death (167). The sustained microglial immune response after the long-term consumption of a hypercaloric diet leads to hypothalamic injury and dysfunction, indicating that the relationship between ARC microglia and neurons is essential for preventing obesity. Taking into consideration the chemokines’ role in obesity-derived hypothalamic microglia activities, Dorfman et al. demonstrated that male mice fed an HFD for 18 weeks presented a reduction in the hypothalamic expression of the neuron-microglia binding protein CX3CL1 (fractalkine) and the mRNA levels of its receptor CX3CR1 (121).

CX3CL1 is a crucial axis for neuron-microglia communication (58, 62, 168). Dissociation of the contact established through CX3CL1 and its receptor promotes microglial pro-inflammatory response (169). I.c.v. CX3CL1 administration significantly suppressed food intake after 48 hours of fasting, while i.c.v. CX3CL1 and NPY co-administration prevented NPY-induced food intake (170). In contrast, maintaining CX3CL1-mediated microglial-neuronal interactions protects against diet-induced obesity (170), highlighting the importance of preserving the relationship between neurons and microglial cells to prevent obesity caused by dietary factors. Future studies should identify the molecular mechanisms involved in the hypothalamic neuron-microglial relationship between health and obesity. In addition, studies should focus on determining the exact moment this interaction is disrupted during a hypercaloric diet since identifying this specific moment might provide information regarding possible targets to restore this interaction and revert the metabolic impairments caused by obesity.

Additionally to the CX3CL1 role, other chemokines are also involved in hypothalamic neuroinflammation. CXCL12 is a chemokine that shows neuroactive effects by promoting the migration of dopaminergic neurons in the midbrain through the Akt-1/FOXO3a axis (171) and modulating electrical excitability in hypothalamic neurons through CXCR4, one of the CXCL12 receptors (172). HFD-fed rats increased expression of CXCL12 and its receptors CXCR4 and CXCR7, which correlated with cognitive decline and locomotor dysfunction (173). CCL2, also known as MCP-1, is produced by microglial cells after an inflammatory stimulus and has been associated with chemoattraction of monocytes in response to acute and chronic inflammatory responses through JAK2/STAT 3, MAPK, and PI3K Pathways (174). Peripheral myeloid cells can be recruited to ARC in hypercaloric diet conditions by crossing fenestrated blood vessels and the third ventricle, which has been related to hypothalamic microgliosis using the CCL2/CCR2 axis in obesogenic diet rodent models (175). CCL2 treatment attracted peripheral macrophage-like cells, and promoted microglial migration, and enhanced CCL2 and proinflammatory cytokine production (176).

As mentioned, cytokines and chemokines are not the only molecules involved in metainflammation and hypothalamic microglial responses. Adipokines have also activity over microglia function since it described that increased leptin, adiponectin, and resistin are correlated with metabolic dysfunction, decreasing food intake and increasing energy expenditure and insulin resistance [reviewed in Recinella et al. (177)]. The effects of the most relevant adipokines on the microglia function are described in **Table 2**.

**Table 2:** Summary of known adipokines and their relationship with microglia *in vitro* and/or *in vivo*.

Adipokines	Effect on microglia	References
Anti-inflammatory adipokines		
**Adiponectin**	Intraperitoneal administration of adiponectin suppressed fatty acid-derived hypothalamic neuroinflammation by modulating COX-2, Iba1, CD11b, IL-1β, IL-6, and TNFα expression.	Song et al. (178)
**Apelin**	Exposition to apelin-13 preserved CD16/32, CD206, iNOS, Arg-1, IL-10, IL-6, and TNFα basal levels ameliorating LPS-induced BV-2 microglia pro-inflammatory response through inhibiting H3K9ac and promoting autophagy.	Peng et al. (179)
**CTRPs**	CTRP4 decreased food intake, suppressed NF-kB signaling and microglial activation *in vivo*, and decreased IL-6 and TNFα production while inhibiting the NF-κB pathway in BV-2 cells.	Ye et al. (180)
**Nesfatin-1**	Nesfatin-1 reduced microglia proinflammatory activation by decreasing IL-1β, IL-6, and TNFα expression in a rat ischemia model.	Erfani et al. (181)
**Omentin-1**	Exposition to recombinant omentin-1 in microglial cell culture suppressed proinflammatory activation, while its depletion increased IL-1β, IL-6, and TNFα cytokine levels.	Ji et al. (182)
**PAI-1**	Plasminogen activator inhibitor type 1 (PAI-1) promoted the migration of microglial cells in culture via the LRP/JAK/STAT1 axis and inhibited microglial engulfment of zymosan particles.	Jeon et al. (183)
**SPARC**	Secreted protein acidic rich in cysteine (SPARC) regulated microglial expansion, branch extension and microglia activation.	Lloyd-Burton et al. (184)
Proinflammatory adipokines		
**Chemerin**	Through Chemerin/CMKLR1 pathway microglia enhanced IL-6 and TNFα production, which was reversed by using α-NETA, an antagonist of CMKLR1.	Yun et al. (185)
**FAM19A5**	Unless there are lack of information of obesity-derived increase of FAM19A5 on microglia function, a knockdown model of FAM19A5 expression resulted in decreased TNFα levels. Also was described as a chemokine which induces hypothalamic inflammation.	Kang et al. (186)
**FSTL1**	A knockdown FSTL1 mouse model inhibited microglia activation through the TLR4/MyD88/NF-κB pathway.	Xiao et al. (187)
**LCN2**	Lipocalin-2 (LCN2) is produced by pro-inflammatory activated microglia through NF-κB signaling.	Jung et al. (188)
**Leptin**	Leptin-stimulated microglia enhanced a pro-inflammatory secretion profile through the ObRb leptin receptor. In rat primary microglial culture leptin induced IL-1β production via STAT3 activation. In mice primary hypothalamic microglia, leptin induced IL-1β, and TNFα, but not Iba1 expression.	Fujita et al. (189) Pinteaux et al. (190) Gao et al. (191)
**RBP4**	Retinol-binding protein 4 (RBP4) activate microglia enhancing Iba1 expression.	Xu et al. (192)
**Visfatin**	Exposition to visfatin in BV2 microglial cells promoted an elevated release of MCP-1, TNFα, IL-6, and IL-1β.	Tu et al. (193)

Although the literature has explored the topic deeply, the mechanisms underlying microglial interactions in obesity remain unclear and require further studies.

## 7 Conclusion

Microglial function under physiological conditions is crucial for maintaining brain homeostasis. Even though not many studies describe the physiological role of microglia in the hypothalamus, it is clear that these cells respond to neuronal activity by rearranging themselves towards these activated neurons, suggesting that these cells play a role in maintaining adequate neuronal functioning in the hypothalamic area. The relationship between these two cell types becomes evident during a hypercaloric diet, where microglial cells surround ARC-POMC neurons and secrete pro-inflammatory molecules like TNFα. Future studies should describe 1) how ARC microglia sense neuronal activity, 2) the functional implication of the neuron-microglial associations, and 3) how these associations become dysregulated during metabolic impairments such as obesity.

Understanding the nature and physiological implication of the relationship between the ARC neuronal populations and microglial cells during health might contribute to identifying therapeutic targets aimed at maintaining this connection even under pathological conditions such as obesity.
